# Knowledge and Attitudes about Organ Donation among Patient Companion at a Tertiary Hopsital in Ethiopia

**DOI:** 10.4314/ejhs.v31i1.14

**Published:** 2021-01

**Authors:** Mahteme Bekele, Wubshet Jote, Tigist Workneh, Berhanu Worku

**Affiliations:** 1 Department of Surgery, St. Paul's Hospital Millennium Medical College, Addis Ababa, Ethiopia; 2 Department of internal Medicine, St. Paul's Hospital Millennium Medical College

**Keywords:** Knowledge, Attitude, organ donation, patient companion

## Abstract

**Background:**

Worldwide, the supply of organs continues to be much less than the demand. Many studies identify poor knowledge and negative attitude about organ donation for this. The purpose of this study was to assess knowledge and attitude towards organ donation and associated factors among people who accompany patient during hospital visit at tertiary hospital in Ethiopia.

**Methods:**

A hospital-based cross-sectional study was conducted from March 1st to May 31, 2019, at a tertiary hospital and the only transplant center in Ethiopia. The patient companions were interviewed with structured questionnaires. Descriptive statistics were presented and factors associated with good knowledge and favorable attitude were assessed using Binary logistic regression.

**Results:**

A total of 414 patient companion participated in the study. The mean age of the respondents was 35±13years, and 252(60.9%) were males. Forty-four (10.6%) of the study participants had good knowledge about organ donation. Male gender was significantly associated with improved knowledge (AOR=2.127 95% CI: 1.036, 4.368). A favorable attitude towards donation was found in 219(52.9%) of the study participants. Participants who had completed secondary education were more likely to have unfavorable attitude towards organ donation (AOR =0.498, 95% CI: 0.290, 0.857).

**Conclusion:**

Knowledge about organ donation was found to be poor, and attitudes towards organ donation were found to be unfavorable among patient companions at a major medical center in Ethiopia. Strategies focused on education of the general public and broad dissemination of information on the benefits of organ donation will be critical for improving the organ donor pool.

## Introduction

Organ donation can be defined as the removal and transfer of biological tissue or an organ of the human body legally from a living person through consent or from a dead person via ascent from next of kin to a living recipient in need of transplantation (1). The history of organ transplantation began in the medicine of mythology. However successful transplantation performed in the late 20^th^ century remains one of the greatest scientific advances and most challenging and complex fields of modern medicine (2–4). The transplant practice reached the African continent in 1967, but the progress is limited to only few countries in the continent (5).

A live person can donate an organ or part of it, but a diseased donor can donate several organs that salvage up to 50 people. Transplantation of the kidney, liver, pancreas, intestine, heart, and lungs has now become a common practice in all parts of the world. An enormous difference has been created between organ supply and demand for donor organs because of increase in the incidences of organ failures and lack of supply of organs. In fact, the rate limiting factors in the current transplantation world is no more surgical technique or rejection, but organ donors (6,7).

Kidney is the most common solid organ transplanted worldwide. In 2006, about 27,000 related and unrelated legal living donor kidney transplants were performed worldwide with 62% of countries reporting at least a 50% increase over a decade (8). Even if organ donation saves life, it is not without risk- the risks may range from postoperative pain to loss of life (9,10).

Overall, organ shortage relative to the increasing demand is a global problem. For instance, the current cadaver organ donation rate in India is 0.08 per million while Spain leads the world with 35 per million (6,11). In addition to the burden of infectious diseases and significant budget cuts in the public health sectors, lack of knowledge, negative attitude, cultural and religious beliefs of the public and professionals involved in the organ donation procedure have negatively impacted the organ donor pool (12–14). A study done on corneal donations demonstrated that the general population has moderate knowledge about donation with fewer people actually pledging donations to the program. This suggests the presence of a large number of unaddressed potential donors (15).

In Ethiopia, kidney transplant started in the year 2015 and the center continued to perform transplantation every month since then (16). A recent study on the knowledge and attitude among medical students about organ donation reported an unfavorable attitude despite good knowledge (14). This study aims to assess knowledge and attitudes about organ donation amongst patients' companions at a major medical center in Ethiopia. The findings from this study will be valuable in informing policies and strategies targeted at improving living and cadaver organ donations.

## Methods and Subjects

A hospital-based cross-sectional study was conducted from March 1 to May 31, 2019, at Saint Paul's Hospital Millennium Medical College (SPHMMC), Addis Ababa, Ethiopia. SPHMMC is a rapidly expanding medical college with a tertiary level healthcare hospital. While the inpatient capacity is slightly over 700 beds, the college sees an average of 1200 emergency and outpatient cases daily. The hospital provides services in various specialty fields including a kidney transplantation service which was started in 2015. The kidney transplant center has one transplant nephrologist, five nephrologists, four transplant surgeons, 20 nurses, and other supportive staffs with a functioning 15 bed dialysis unit, 4 ICU and ward beds, 24 hour working pharmacy and laboratory. The center so far performed more than 140 kidney transplant and continues to perform 4 transplants every month.

The study population included all patient companions who were above 18 years of age and accompanying patients to various out patients and inpatient departments of the hospital from March 1 to May 31, 2019. Patient companion whose patients were critical and required immediate hospitalization, companions of patients who were to undergo organ transplantation surgery and those who were frustrated, unstable or incapable of responding to survey questions were excluded

The sample size was determined using single population proportion formula by considering the following statistical assumptions: 95% confidence interval (CI), 50% proportion (as proportion could not be taken from previous studies) and 5% marginal error. Considering 10% for non-response rate, the final sample size for this study was 422. Simple random sampling method was employed to select the study participants.

The knowledge questionnaire focused on the organ donation, the presence of organ registry in Ethiopia, the age limit for organ donation, possibility of donating whole or part of an organ, there risk and benefit of donating an organ, Ethiopian organ donation law and access to transplant service. The selected knowledge questions responses were summed up and rated out of 12.

The attitude questionnaire focused on the participants' attitude towards willingness to donate, the value of organ donation in saving lives, registration as donor if national registration is available, donation after death, promoting organ donation and willingness to explore more about organ donation scientific and religious view. Each question was rated out of five on Likert's scale and finally summed up to get the final score out of 35. The following cut of point was used to measure the respondents' knowledge and attitude about organ donation. **Knowledge**: understanding about organ donation **Good**- participants who scored ≥ 75% on knowledge based questions and **Poor**- participants who scored < 75% on knowledge based questions. **Attitude**: a feeling or emotion toward organ donation. **Favorable**participants who scored ≥ 75% on attitude based questions. **Unfavorable**- participants who scored <75% on attitude based questions.

The study was conducted after ethical clearance was obtained from SPHMMC Institutional Review Board. Written informed consent was obtained from the participants. The objectives and importance of the study were explained to the participants before the interview. Participants were notified that their participation was voluntary and that they could withdraw at any time during the data collection process. Participants' confidentiality, privacy and anonymity were maintained throughout the study period.

Data was collected by trained general practitioners using a structured questionnaire which had been tested and corrected before the actual data collection. Four trained general practitioners collected the data by face-to-face interview of patient companion at the different outpatient departments of the hospital. The collected data was checked for completeness coded, entered into Epi-Info version 7.2.1.0, and exported to SPSS version 23.0 software for analysis. Participants' socio-demographic characteristics, source of information, knowledge and attitude are presented using the relevant descriptive statistics. The association between the dependent variable and independent variables was analysed using Binary Logistic Regression and Multiple Logistic Regression. And, 95% confidence interval was calculated and variables with p-value ≤ 0.05 were considered as statistically significant.

## Results

**Socio-demographic characteristics**: Among 422 patient companions, 414 responded the entire questionnaire providing a response rate of 98.1%. As outlined in Table 1, the mean age of the participants was 35±13, and 176 (42.5%) were in the age range of 18–29 years. More than half of the study participants were males with male-to-female ratio of 1.6:1. The majority of the study participants were married (60.4%). One hundred thirty (31.4%) of the study participants were degree holders and postgraduates. Regarding occupation, 171(41.3%) of the respondents were private employees. Close to two third (64.0%) of the respondents were from Addis Ababa. Two hundred twenty-six (54.6%) of the participants were orthodox Christians.

**Table 1:** Socio-demographic and source of information variables among Patient companion, Addis Ababa, 2019 (n=414)

Variable	Frequency	Percentage (%)	Variable	Frequency	Percentage (%)
**Age group (in years)**			**Education**		
18–29	176	42.5	No formal education	44	10.6
30–39	119	28.7	Primary school complete	66	15.9
40–49	58	14.0	Secondary school complete	103	24.9
>=50	61	14.7	Diploma holder	71	17.1
**Sex**			Degree holder and postgraduate	130	31.4
Male	252	60.9	**Occupation**		
Female	162	39.1	Student	74	17.9
**Place of residence**			Government Employee	90	21.7
Addis Ababa	265	64.0	Private Employee	171	41.3
Outside Addis Ababa	149	36.0	Unemployed	44	10.6
			Others	35	8.5
**Religion**			**Source of information**		
Orthodox	226	54.6	Television	353	85.3
Muslim	101	24.4	Word of mouth (Friends and Colleagues)	299	72.2
Protestant	81	19.6	Health care facilities	195	47.1
Catholic	5	1.2	Radio	157	37.9
Not mentioned	1	0.2	Internet/online source/ social network	131	31.6
**Marital status**			Movies	114	27.5
Single	145	35	Newspaper	44	10.6
Married	250	60.4	Posters	36	8.7
Divorced or widowed	19	4.6	Organ donation promotion campaigns	30	7.2
Total	414	100	Others	57	13.8

The largest sources of information about organ donation among the respondents were television programs 353(85.3%) and word of mouth from friends and colleagues 299 (72.2%), (Table 1). Three hundred fifty-six (86%) of the respondents claimed that they got information about organ donation from more than one source.

**Knowledge about organ donation**: Almost half of the study participants, 220(53.1%), answered that organ donation means the transfer of tissues/blood/organs from a living or a dead body to a patient in need. Almost all of the study participants knew at least one organ that can be donated.

Regarding the type of organ that can be donated, the majority of the participants mentioned kidney (90.3%) followed by blood (85.7%). On the other hand, only two individuals (0.5%) mentioned that the pancreas can be donated. Three hundred fifty-one (84.8%) respondents correctly mentioned more than one organ that can be donated. In response to the question “Does your religion allow organ donation”, 170(41.1 %) answered yes”’ (Table 2). More than half of the participants knew that there is a donor registry in Ethiopia where people register to donate organs after death 285(68.8%) and that the legal donation age is 18 years and above, 233(56.3%). Also, the majority of the participants knew that Ethiopia's organ donation law and policy prohibits buying or selling of organs, 245(59.2%), and 269(65%) knew that transplant centers provide access for all nationalities equally (Table 2).

**Table 2:** Responses for knowledge-based questions about organ donation among Patient attendants, Addis Ababa, 2019 (n=414)

Variable				Frequency	Percent (%)
What does organ/tissue/blood donation mean to you?			Transfer of tissues or organ from a dead body to a patient in need	75	18.2
			Transfer of tissues/blood/organs from a living to a patient in need	119	28.7
			All of the above	220	53.1

Do you know organ can be donated			Yes (one or more organ mentioned)	413	99.8
			I don't know	1	0.2

What organs tissues can be donated					
Kidney	374	90.3	Bone marrow	49	11.8
Blood	355	85.7	Skin	41	9.9
Cornea of the eyes	193	46.6	Bone	22	5.3
Liver	123	29.7	Lungs	13	3.1
Heart	56	13.5	Pancreas	2	0.5
			Intestine	8	1.9

There is a donor registry in Ethiopia where people register during their life to donate organs after death. Have you heard about it?			Yes	285	68.8
			No	129	31.2

At what age can an individual register for organ donation?			At any age	41	9.9
			18 years and above	233	56.3
			do not know	140	33.8

Death could mean			The heart is not beating and there is no breathing	339	81.9
			Brain death in which the heart is beating with the help of ventilator to keep breathing	49	11.8
			I do not know	26	6.3

Does your religion allow organ donation			Yes	170	41.1
			No	119	28.7
			I don't know	125	30.2

Do you know anyone who has donated an organ?			Yes	152	36.7
			No	262	63.3

Do you know that during life an individual can donate a part of his liver to his relative			Yes	123	29.7
			No	291	70.3

Do you know that donating a part of your liver is a risk to your health			Yes	166	40.1
			No	123	29.7
			May be	42	10.1
			Don't know	83	20.0

Do you know that you can donate one of your two kidneys during your life, to another person			Yes	374	90.3
			No	40	9.7

Do you know that donating a kidney is safe			Yes	116	28.0
			No	218	52.7
			May be	62	15.0
			Don't know	18	4.3

Do you know the Ethiopia's organ donation law and policy Prohibits any buying or selling of organs			Yes	245	59.2
			No	169	40.8

Do you know the Ethiopia's organ donation law and policy provides access to transplant facility for all nationalities equally			Yes	269	65.0
			No	145	35.0

Forty-four (10.6%: 95% CI: 7.5, 13.5) participants were found to have good knowledge about organ donation, and the remaining 370 (89.4%: 95% CI: 86.5, 92.5) were rated as having poor knowledge.

**Attitude towards organ donation**: Among the participants, 219(52.9%) responded strong agreements towards promoting organ donation while only 106(25.6%) expressed their strong agreement to register as organ donor (Table 3). On the final score, 219(52.9%: 95% CI: 47.9, 57.5) had favorable attitudes towards organ donation, and 195(47.1%: 95% CI: 42.5, 52.1) reported unfavorable attitudes.

**Table 3:** Responses for attitude related questions towards organ donation among Patient attendants, Addis Ababa, 2019 (n=414)

Attitude question	Strongly agree	Agree	Neutral	Disagree	Strongly Disagree
Organ donation is a good thing and should be	219	156	19	16	4
promoted	(52.9)	(37.7)	(4.6)	(3.9)	(1.0)
Registering as organ donor could save somebody's	188	192	15	17	2
life	(45.4)	(46.4)	(3.6)	(4.1)	(0.5)
Any Ethiopian citizen should be automatically	73	158	93	79	11
included on the organ donor register of Ethiopia,	(17.6)	(38.2)	(22.5)	(19.1)	(2.7)
with the ability to refuse if they wish					
I am willing to register as an organ donor, if my	106	134	72	83	19
family would have no objection to allowing donation	(25.6)	(32.4)	(17.4)	(20.0)	(4.6)
of my organs at the time of my death					
I am willing to register as an organ donor, If I knew	87	167	63	80	17
more about what is organ transplant and how it is	(21.0)	(40.3)	(15.2)	(19.3)	(4.1)
done					
I am willing to register as an organ donor, If more	95	168	53	84	14
information was available about the view point of	(22.9)	(40.6)	(12.8)	(20.3)	(3.4)
my religion with regard to organ donation					
I am willing to register as an organ donor, If I knew	73	144	67	103	27
where I could register	(17.6)	(34.8)	(16.2)	(24.9)	(6.5)

**Factors Associated with knowledge and attitude of organ donation**: From univariate analysis of the independent variables, sex, place of residence, education and attitude towards organ donation were significantly associated with good knowledge about organ donation among patient companions, at 25% level of significance. However, only sex was found to be significantly associated with good knowledge about organ donation among patient companions in the Multiple Logistic Regression Model at 5% level of significance.

Accordingly, the odds of having good knowledge about organ donation among male patient companions was 2.127 times the odds of having good knowledge about organ donation among female patient companions (AOR=2.127 95% CI: 1.036, 4.368). Details of factors associated with knowledge about organ donation among patient companions are presented in Table 4.

**Table 4:** Univariate and Multivariate analysis for factors associated with knowledge about organ donation among Patient companion, Addis Ababa, 2019 (n=414)

Variables	Knowledge		COR(95% Cl)	AOR(95% Cl)	p-value
				
	Good	Poor			
**Sex**					
Male	32	220	1.818 (0.907, 3.644)	**2.127 (1.036, 4.368)**	0.040*
Female	12	150	1.00	1.00	
**Place of residence**					
Addis Ababa	32	233	1.568(0.782,3.145)	1.371(0.664,2.831)	0.393
Outside Addis Ababa	12	137	1.00	1.00	
**Education**					
No formal education	1	43	0.136(0.018, 1.046)	0.159(0.020, 1.259)	0.082
Primary school complete	5	61	0.479(0.170, 1.346)	0.416(0.144, 1.198)	0.104
Secondary school					
complete	13	90	0.844 (0.395, 1.801)	0.864 (0.399, 1.870)	0.710
Diploma holder					
Degree holder and post	6	65	0.539(0.205, 1.419)	0.535(0.199, 1.435)	0.214
graduate	19	111	1.00	1.00	0.210
**Attitude**					
Favourable	27	192	0.679 (0.358, 1.288)	0.759 (0.391, 1.474)	0.416
Unfavourable	17	178	1.00	1.00	

**Note:** COR, Crude odds ratio; AOR, Adjusted odds ratio; CI, Confidence interval;

* Statistically significant

From univariate analysis of the independent variables, place of residence, more than one source of information, religion, education and knowledge about organ donation were significantly associated with favourable attitudes towards organ donation among patient companions at 25% level of significance. However, only more than one source of information and education were found to be significantly associated with favourable attitudes towards organ donation among patient companions in the Multiple Logistic Regression Model at 5% level of significance.

The odds of having favourable attitude towards organ donation among patient companions who had more than one source of information regarding organ donation were 2.2 times the odds of those with only one source of information (AOR=2.153 95% CI: 1.161, 3.993).

Regarding level of education, patient companions who had completed secondary school and those who were diploma holders were 50.2% and 63.3% times less likely to have favorable attitude towards organ donation compared to those who were degree holders and had postgraduate levels of training (AOR=0.498, 95% CI: 0.290, 0.857 for secondary school complete and AOR=0.367, 95% CI: 0.198, 0.680 for diploma holders). Details of the factors associated with attitude towards organ donation among patient companions are presented in Table 5.

**Table 5:** Univariate and Multivariate analysis for factors associated with attitude towards organ donation among Patient companion, Addis Ababa, 2019 (n=414)

Variables	Attitude		COR(95% CI)	AOR(95% CI)	p-value
	**Favourable**	**Unfavourable**			
**Place of residence**					
Addis Ababa	151	114	1.578 (1.054,2.363)	1.419 (0.916,2.199)	
Outside Addis Ababa	68	81	1	1	0.117
**More than one source of information**					
Yes	200	161	2.223 (1.222,4.045)	2.153 (1.161,3.993)	0.015*
No	19	34	1	1
**Religion**					
Orthodox	129	97	1.425 (0.868,2.340)	1.600 (0.942,2.720)	0.082
Muslim	48	53	0.970 (0.547,1.723)	0.999 (0.549, 1.818)	0.997
Others	42	45	1	1	0.084
**Education**					
No formal education	19	25	0.402 (0.200,0.808)	0.489 (0.233,1.025)	0.058
Primary school complete	36	30	0.635 (0.347,1.163)	0.653 (0.349,1.220)	0.182
Secondary school complete	50	53	0.499 (0.294,0.848)	0.498 (0.290,0.857)	0.012*
Diploma holder	29	42	0.366 (0.202,0.663)	0.367 (0.198,0.680)	0.001*
Degree holder and post graduate	85	45	1	1	
**Knowledge**					
Good	27	17	0.679 (0.358,1.288)	0.745 (0.382,1.453)	0.387
Poor	192	178	1	1	

**Note**: COR, Crude odds ratio; AOR, Adjusted odds ratio; CI, Confidence interval

* Statistically significant

## Discussion

The major concern of every transplantation program is the availability of donor organ, and different countries have taken different measures to promote donation among citizens. Despite these efforts, the number of available organs is still below the requisite and professionals indicated that the plausible reasons for this shortage include the public's insufficient awareness.

This study provided analysis of the Ethiopia patient companions' knowledge and attitude about organ donation. The mean age of the participants was 35+14 years which is similar to the study conducted in patient relatives of Emergency Department of Iran and regular Outpatient Department in Bengaluru, India (17, 18).

Globally, the prevalence of knowledge of organ donation ranges from 60 to 85%, which varies between countries (19). Knowledge assessment of our study revealed that almost all study participants heard of organ donation, which is encouraging and comparable to the Saudi Arabia study of 98.3% and slightly higher than 89.1% and 86% in Pondicherry and Ahmadabad, India, respectively (20,21,22). It is quite higher than a study done in Nigeria which showed that 60% respondents heard of the term organ donation (23). The result of our study is even comparable to a study conducted among medical students of St. Paul's Hospital Millennium Medical College, where 98.3% of the participants heard of organ transplantation (14). However, only small percentage (10.6%) of our study participants were knowledgeable about organ, which is much lower compared to population-based studies in Pakistan (25.5%) and South India (28%), and also lower than relatives of patients in the Emergency Department of Iran who scored 34.2% (17,19,24). It is far below the global estimate of prevalence of 60–80% knowledge which varies from country to country (19).

Kidney was mentioned as the organ that can be donated by 90.7% of the participant in this study, which is comparable to the study in Saudi Arabia where 95.5% of the participants mentioned kidney as an organ to be donated (20). This may be resulted from the existing practice of kidney transplant in the country and dissemination of information in the different media.

Media played a great role as source of information, and television advertising is one of the effective way of communicating information about organ donation. In our study, the major source of information mentioned by the participants was TV, which is the commonest source of information about organ donation among the general population and medical professionals (14,17,18). The media shall be seriously recognized by the scientific community as the powerful tool that can influence the public both in a positive and a negative sense (25).

The male gender was identified as a factor that was significantly associated with good knowledge about organ donation among patient companions. This is in agreement with a general population based study conducted in India that found males being aware of organ donation than females (26). This might be resulted from a better access to information and adequate time to explore the media by males than females, as females has more household responsibility in developing countries. However, a study conducted on patient companions in Iran and a population based study in Nigeria, found female gender associated with good knowledge (17, 23).

Nearly half of the study participants had favorable attitude towards organ donation. These figures are lower than the studies done on African Americans in the US, and much lower than the percentage of black South Africans who were willing to donate their own organs (76%) (27, 28). As transplantation is new in Ethiopia, a lower rate of favorable attitude towards organ donation is expected.

As for factors associated with attitude towards organ donation among patient companions, the study identified more than one source of information and higher levels of education as significantly associated factors. A study done in India on the general population revealed that only about 21% became aware through mass media. The largest source of information found in this study was television as part of the mass media showing the already growing media platform for further awareness creation (26). This may be due to the fact that repeated, multiple and different exposure to information might affect the way we judge and feel about things giving directions for possible public campaign media outlets

Patient companions who had completed secondary school and held diplomas were less likely to have favorable attitude towards organ donation compared to those who were degree holders and had postgraduate training. These findings are also reflected in a study done in North West Ethiopia on eye donation (15). This may be due to the fact that education improves the way individuals understand and perceive things, providing a logical base for decisions and favorable attitudes towards given practices.

The strengths of this study include that it tried to address the issue of organ donation among patient companions, which can produce new evidence about the extent of understanding of the issue in the population. The limitations of the study are the population studied can not exactly represent the entire population requiring result to be cautiously interpreted and that it does not explore the deep reason for poor knowledge and unfavorable attitude.

In conclusion knowledge about organ donation among patient companions at a medical center in Addis Ababa, Ethiopia, was found to be poor and the male gender was found to be a significant factor associated with good knowledge. Nearly half of the patient companions' attitude towards organ donation was found to be unfavorable and having more than one source of information and a higher educational level were found to be significantly associated with a favorable attitude towards organ donation. We recommended awareness creation on the general public about the benefits of organ donation using multiple media platforms and at all educational levels. As the two largest sources of information were television and word of mouth, working on television programs and social media platforms as possible public campaign outlets on this issue will help improve the society's knowledge about this subject. Future work can focus on conducting studies at a community level about knowledge and attitudes about organ donation in order to gather relevant information about the general population.
